# Effects of Functional Limb Overloading on Symmetrical Weight Bearing, Walking Speed, Perceived Mobility, and Community Participation among Patients with Chronic Stroke

**DOI:** 10.1155/2015/241519

**Published:** 2015-10-27

**Authors:** Sami S. Alabdulwahab, Fuzail Ahmad, Harpreet Singh

**Affiliations:** ^1^Department of Rehabilitation Sciences, College of Applied Medical Sciences, King Saud University, Riyadh 11952, Saudi Arabia; ^2^Department of Physical Therapy & Health Rehabilitation, College of Applied Medical Sciences, Majmaah University, Al-Majmaah 11451, Saudi Arabia; ^3^Department of Neurology & Neurosurgery, All India Institute of Medical Sciences, New Delhi 110029, India

## Abstract

*Background*. Stroke is a leading cause for long-term disability that often compromises the sensorimotor and gait function accompanied by spasticity. Gait abnormalities persist through the chronic stages of the condition and only a small percentage of these persons are able to walk functionally in the community. *Material and Method*. Patients with chronic stroke were recruited from outpatient rehabilitation unit at Department of Neurology & Neurosurgery, All India Institute of Medical Sciences, having a history of first stroke at least six months before recruitment, with unilateral motor deficits affecting gait. The patients were randomly assigned to either the functional limb overloading (FLO) or Limb Overloading Resistance Training (LORT) group and provided four weeks of training. *Result*. We found that there was an improvement in gait performance, weight bearing on affected limb, and perceived mobility and community participation. *Conclusion*. To the best of our knowledge, this is the first study that has evaluated the effects of functional limb overloading training on symmetric weight bearing, walking ability, and perceived mobility and participation in chronic hemiplegic population. The study demonstrated a beneficial effect of training on all the outcomes, suggesting that the functional limb overloading training can be a useful tool in the management of gait problems in chronic stroke patients.

## 1. Introduction

Stroke is becoming a rapidly increasing problem and an important cause of disability and deaths worldwide. Incidence and prevalence of strokes in Saudi Arabia are comparatively lower than western countries, which could be because of the predominance of the younger age groups in this region [1]. The annual stroke incidence ranged from 27.5 to 63 per 100,000 population and prevalence ranged from 42 to 68 per 100,000 population [2].

Stroke is a leading cause for long-term disability due to compromised sensorimotor function. Approximately 85% of stroke survivors learn to walk independently by 6 months after stroke, but gait abnormalities persist throughout the chronic stages of the condition. Only a small percentage of stroke survivors are able to walk functionally in the community [3, 4].

The objective of stroke rehabilitation is to enable individual patients to maximize benefits from training in order to attain the highest possible degree of physical and psychological performance. The ultimate goals for many stroke patients are to achieve a level of functional independence necessary for returning home and to integrate as fully as possible into community life.

Ng and Hui-Chan [5] have noted that weakness in hemiplegic stroke patients is sometimes overshadowed over concerns about treatment of spasticity and synergistic movements. Studies have revealed positive correlations between the strength of specific muscle groups and a variety of functional attributes [6]. Furthermore, a nonlinear relationship between walking performance and muscle strength in the lower extremities has been suggested [7]. However, as the protocols were multifaceted, it was not possible to determine the precise role that the strength-training component may have played in improving walking function.

A number of studies have shown that task specificity and intensity of training are the main determinants of functional improvement after stroke [6, 8, 9]. Moreover, there is growing evidence suggesting that intensive task-oriented practice can induce greater improvement in walking competency than usual practice in stroke survivors [10–12].

Yang et al. [13] in their study on stroke patients undergoing progressive lower limb strengthening using functional weight bearing activities found moderate increases in walking speed. Sullivan et al. [14] found that task-specific training with body-weight support is more effective in improving walking speed but lower limb strength training did not provide any added benefit to walking outcomes.

A major limitation to the conclusions from these studies and systematic reviews is the lack of consistency in the intervention and specified protocols [15–17]. Despite the number of studies dedicated to task-oriented training, none of these studies had combined functional task training with prolonged resistance in the form of limb overloading applied 90% of the awake time. Therefore, we designed this study to address the evidence related to our training protocol to enhance symmetric weight bearing and walking speed and its impact on perceived mobility and community participation in patients with chronic stroke.

We hypothesized that intervention programs that combine lower limb overload with functional task training would be more effective at improving walking outcomes and community participation than lower limb overload training alone.

The design of our study was influenced by the literature on lower extremity strength training and task-specific locomotor training [14, 18, 19]. The use of this design should provide valuable comparisons that reveal the practical benefits of functional limb overloading training to improve walking outcomes after stroke and its effect on perceived community participation.

## 2. Material and Method

Chronic stroke patients were recruited from outpatient rehabilitation unit at Department of Neurology & Neurosurgery, All India Institute of Medical Sciences. Patient having; (a) history of first stroke at least six months before recruitment, with unilateral motor deficits affecting gait pattern and/or speed, (b) independent walking ability with or without walking aid for at least 10 m, and (c) ability to perform closed-chain exercise were selected for the study. Exclusion criteria included significant psychiatric or cognitive deficits, major cardiorespiratory diseases, chronic pain, or lower limb joints complication.

Study protocols were explained to patients and written informed consent was obtained from them. The patients were then randomly assigned to either the functional limb overloading (FLO) group or Limb Overloading Resistance Training (LORT) group by drawing one of two sealed envelopes designating the group membership.

## 3. Management Protocol

The patients in FLO group underwent task-oriented gait training of one-hour duration three times per week for four weeks. They were requested to wear the weight cuff, equivalent to 5% of the total body weight, for most of their awake time (approximately 90% of the time). Brown et al. [18] in their study used 15% of the body weight for limb overloading during a cycling task. As our study required the patients to wear weighted cuff most of their awake time and to perform functional tasks wearing these cuffs, therefore, after a pilot trial on few patients, we settled for 5% of the body weight (3–5 Kg), which patients tolerated without much distress.

During the training session, after 5–10 minutes of simple warm-up exercises, the patients performed various functional activities including forward, sideways, and backward walking, turning to 90 and 180 degrees during walking, stair climbing, and so forth. During each session the patients were also made to walk on treadmill for 15 minutes at speed of 10% above comfortable walking. The progression of the training was done by increasing the loading or steps height, or by reducing the speed, or decreasing the patient's support and/or doing more individually tailored sets, but as a rule the difficulty level was adjusted after six sessions.

The patients in the LORT group underwent resistance-training regime consisting of isotonic exercise using weight cuff tied to ankle or foot. The exercise algorithm was designed by the therapist accounting for strength as well as movement synergy level, to determine repetition maximum (RM) with the given load. The training protocol required each participant to isotonically exercise the affected lower extremity (LE) using external resistance of weight cuff, equivalent to 5% of the total body weight. The training session consisted of 5–10 minutes of simple warm-up exercises and then three sets of 10–15 repetitions maximum, to moderate fatigue with limb overloading. The therapist follows an exercise algorithm that accounted for the participant's strength as well as movement synergy level to determine RM for specific muscle groups, including hip flexors, hip extensors, knee flexors, knee extensors, ankle dorsiflexors, and ankle plantar flexors, with the given weight.

The outcome measures taken for this study were Cadence, Fast Gait Speed (FGS), Slow Gait Speed (SGS), weight bearing on affected limb (WBAL), and perceived mobility and participation of stroke patients.

The Walkway gait analysis system (TekScan, USA) was used to capture plantar pressure data and temporal (time) and spatial (distance) gait parameters. This system consists of a 5 mm thickness of floor mat composed of 2,288 resistive sensors, with a resolution of 1.4 sensors/cm^2^, a sensor matrix measuring 439.5 mm by 369.9 mm, and a sampling frequency of 40 Hz.

Perceived mobility and participation were assessed by the stroke impact scale (SIS) which is a self-report questionnaire that assesses aspects of the impact of a stroke on an individual's self-perceived health [20]. Interrater reliability and concurrent validity have been found to be good for SIS [21]. For our study, we have used only mobility and participation domain of SIS. For each subject, the mean scores of items from mobility and participation section were calculated and converted into percentage. High values represent no or few restrictions in participation and low values indicate participation that is more restricted.

An unequal group pretest-posttest design was used and the group comparisons at baseline and after intervention were analysed using a *t*-test. An alpha level set at 0.05 determined significance in two-sided hypothesis testing.

All analyses were performed using SPSS version 20.0.

## 4. Results

Twenty-six patients participated in this study. Three patients in LORT group dropped out due to motivational reasons.

The mean age of the participants was 45.2 ± 12.5, 78% of them were male, and the remaining 22% were female. About 44% of the participants had left side hemiplegia and 56% of them had right side hemiplegia (Table 1).

All the participants had stroke at least six months priorly with a mean duration of 16 ± 10 months, unilateral motor deficits affecting gait, independent walking ability with or without walking aid, and ability to perform closed-chain exercise.

Table 2 shows the patient characteristics of both groups at baseline. No statistically significant differences between both groups were found with respect to the selected outcome variables. No statistically significant differences were found (*p* < 0.05) with respect to measurement of Cadence, SGS, FGS, WBAL, and SIS.

Cadence showed an improvement of 33% (*t* = 5.03,   *p* = 0.001^*∗*^) and 28% (*t* = 2.34, *p* = 0.044^*∗*^) in FLO and LOTR group, respectively, which were statistically significant.

The posttest values of SGS suggested an increase of 47% (*t* = 3.62, *p* = 0.004^*∗*^) and 32% (*t* = 2.6,   *p* = 0.029^*∗*^), respectively, in FLO and LOTR groups. However the Fast Gait Speed (FGS) improved by 34% (*t* = 4.68,   *p* = 0.001^*∗*^) and 21% (*t* = 2.28, *p* = 0.049^*∗*^), respectively, in the FLO and LOTR groups. These improvements were found to be statistically significant (Tables 3 and 4).

**Figure 1:**
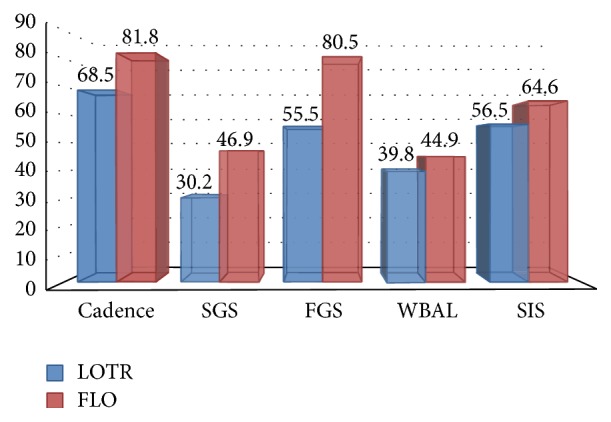
Posttest intragroup comparison of outcome variables. SGS: Slow Gait Speed, FGS: Fast Gait Speed, WBAL: weight bearing on affected limb, and SIS: stroke impact scale score.

To find the difference between both groups an independent *t*-test was used which showed 19.4% (*t* = 2.45,   *p* = 0.024^*∗*^) improvement in Cadence, 55.3% (*t* = 2.81,   *p* = 0.011^*∗*^) improvement in SGS, 45% (*t* = 2.47,   *p* = 0.023^*∗*^) improvement in FGS, 12.8% (*t* = 2.19,   *p* = 0.040^*∗*^) improvement in WBAL, and 14.3% (*t* = 2.27,   *p* = 0.046^*∗*^) improvement in SIS scores after training (Table 5, Figure 1).

Posttraining weight bearing on affected limb improved significantly by 11% (*t* = 2.75,   *p* = 0.018^*∗*^) in FLO group, while very little improvement in LORT group was detected.

Perceived mobility and community participation significantly improved to 16% in the FLO group (*t* = 2.69, *p* = 0.020^*∗*^). In contrast, they showed insignificant improvement (6%) in LORT group (*t* = 1.71,   *p* = 0.121). (Table 5, Figure 1).

## 5. Discussion

The aim of the present study was to find the efficacy of functional limb loading training on gait performance, weight bearing on affected limb, and perceived mobility and community participation in chronic community dwelling stroke patients. In our study we found an improvement in all the selected variables. In patient with chronic stroke, the common impairments associated with muscle strength, motor control, and balance appear to have the strongest relation with walking [22]; therefore these improvements may be due to the increased physical activity undertaken during the training period as it is well documented that patients with chronic stroke have relatively sedentary lifestyle with little physical activities [23]. In the present study, the four weeks of training resulted in symmetric weight bearing and improved gait function, which may be due to the improved muscle strength, coordination, and sensory organization caused by functional overloading of the affected limb. The lower limb overloading along with functional activities could have increased the weight bearing on the affected leg and the proprioceptive stimuli to the joints of the paretic leg causing increased stability.

The significant improvement in terms of weight bearing symmetry on affected leg could be due to the changes in biomechanical alignment of the body, resulting in equal transfer of body weight to both lower limbs. These results can be explained based on previous studies, which suggest that the hemiplegic patients showing asymmetric lower limb weight bearing generally had associated sensory derangements and proprioceptive stimulation can influence postural control and ambulation in such patients [24]. The complex mechanisms, which contribute to gait, and the varying environments demands during functional mobility require interventions that can addresses different elements underlying walking. Impaired ability of the paretic limb to maintain symmetrical loading affects balance and gait. Hendrickson et al. [25] suggested that rehabilitation strategies that increase the contribution of the paretic limb to standing balance control may increase symmetry of walking after stroke. The improvement shown by the subjects in the FLO group may be due to the continuous loading provided to them, which amounted to approximately 90% of awake time.

Even though the effects of resistance training on gait performance are less clear, various studies have demonstrated strong correlations between walking speed and lower limb muscle strength of the paretic side suggesting its positive effect on gait speed and endurance [26]. The curvilinear relationship between muscle strength and functional skills suggests that further improvements in strength after the achievement of functional level may not lead to any substantive gains. Therefore, the significant improvement in the weight bearing on affected lower limb and gait performance in our study may be because of the fact that limb overloading is incorporated with functional activities directed at improving the gait function. The results of our study substantiate the strong evidence suggesting that task-specific training can assist with functional motor recovery, which is driven by adaptive neural plasticity [27]. In this study, the subjects in functional limb overloading training showed a statistically significant improvement in perceived mobility and community participation. The findings are similar to the study done by Flansbjer et al. [28] assessing the effect of progressive resistance training on perceived participation in stroke patient, which found that improvements in gait performance were related to improvements in perceived participation. Other studies measuring changes in function and disability found that self-reported limitations in performing life tasks decreased following resistance training.

## 6. Conclusion

To the best of our knowledge, this is the first study that has evaluated the effects of functional limb overloading training on weight bearing on affected limb, walking ability, and perceived mobility and community participation in chronic hemiplegic population. The study demonstrated a beneficial effect of training on all the outcomes, suggesting that the functional limb overloading training can be a useful tool in the management of gait problems in chronic stroke patients.

## Figures and Tables

**Table 1:** Descriptive statistics.

	%
Gender	
Female	21.7
Male	78.3
Side affected	
Left side	43.5
Right side	56.5
Duration since stroke onset	
6–12 months	30.4
1-2 years	56.5
2-3 years	13.0

**Table 2:** Between-group comparison of the dependent variable at baseline.

Variables	FLO	LORT	Independent *t*-test	
	*N* = 13	*N* = 10	*t*	*p*
Age	45.3 + 12.3	44.7 + 14.1	0.12	0.911
Cadence	61.1 + 12.2	53.4 + 15.4	0.98	0.161
SGS (m/s)	31.9 + 15.9	22.8 + 11	1.50	0.149
FGS (m/s)	59.8 + 26.2	45.5 + 17.7	1.58	0.131
WBAL	40.2 + 6.9	38.4 + 8.2	0.31	0.759
SIS	55.6 + 7	53.1 + 10.4	0.91	0.376

SGS: Slow Gait Speed, FGS: Fast Gait Speed, WBAL: weight bearing on affected limb, and SIS: stroke impact scale score.

**Table 3:** Within-group comparison of the dependent variable in FLO group.

Variables	Pretest	Posttest	Paired	
	M ± SD	M ± SD	*t*-test	
	*N* = 13	*N* = 13	*t*	*p*
Cadence	61.1 ± 12.2	81.8 ± 13.8	5.03	5.03
SGS (m/s)	31.9 ± 15.9	46.9 ± 15.9	3.62	0.004^*∗*^
FGS (m/s)	59.8 ± 26.2	80.50 ± 30	4.68	0.001^*∗*^
WBAL	40.2 ± 6.9	44.9 ± 3.4	2.75	0.018^*∗*^
SIS	55.6 ± 7	64.6 ± 3.4	2.69	0.020^*∗*^

SGS: Slow Gait Speed, FGS: Fast Gait Speed, WBAL: weight bearing on affected limb, and SIS: stroke impact scale score.

**Table 4:** Within-group comparison of the dependent variable in LORT group.

Variables	Pretest	Posttest	Paired	
	M ± SD	M ± SD	*t*-test	
	*N* = 10	*N* = 10	*t*	*p*
Cadence	53.4 ± 15.4	68.5 ± 14.5	2.34	2.34
SGS (m/s)	22.8 ± 11	30.2 ± 11	2.6	0.029^*∗*^
FGS (m/s)	45.5 ± 17.7	55.5 ± 24.1	2.28	0.049^*∗*^
WBAL	38.4 ± 8.2	39.8 ± 6.9	0.86	0.412
SIS	53.1 ± 10.4	56.5 ± 6.8	1.71	0.121

SGS: Slow Gait Speed, FGS: Fast Gait Speed, WBAL: weight bearing on affected limb, and SIS: stroke impact scale score.

**Table 5:** Posttest between-group comparisons of the dependent variables.

Variables	FLO	LORT	Independent *t*-test	
	*N* = 13	*N* = 10	*t*	*p*
Cadence	81.8 ± 13.8	68.5 ± 14.5	2.45	0.024^*∗*^
SGS (m/s)	46.9 ± 15.9	30.2 ± 11	2.81	0.011^*∗*^
FGS (m/s)	80.50 ± 30	55.5 ± 24.1	2.47	0.023^*∗*^
WBAL	44.9 ± 3.4	39.8 ± 6.9	2.19	0.040^*∗*^
SIS	64.6 ± 3.4	56.5 ± 6.8	2.27	0.046^*∗*^

SGS: Slow Gait Speed, FGS: Fast Gait Speed, WBAL: weight bearing on affected limb, and SIS: stroke impact scale score.
